# Introduction of a psychologically informed educational intervention for pre-licensure physical therapists in a classroom setting

**DOI:** 10.1186/s12909-020-02272-5

**Published:** 2020-10-23

**Authors:** Lindsay A. Ballengee, J. Kyle Covington, Steven Z. George

**Affiliations:** 1 Department of Orthopaedic Surgery, Duke University School of Medicine and Durham VA Health Care System Center of Innovation to Accelerate Discovery and Practice Transformation, 411 W Chapel Hill Street Ste 600, Durham, NC 27701 USA; 2 Department of Orthopaedic Surgery, Division of Physical Therapy, Duke University School of Medicine, 311 Trent Drive, Durham, NC 27710 USA; 3 Department of Orthopaedic Surgery and Duke Clinical Research Institute, Duke University, 200 Morris Street, Durham, NC 27001 USA

**Keywords:** Implementation, Psychologically informed physical therapy, Pain, Education

## Abstract

**Background:**

There is an increasing need for physical therapists to address psychosocial aspects of musculoskeletal pain. Psychologically informed practice is one way to deliver this type of care through the integration of biopsychosocial interventions into patient management. An important component of psychologically informed practice is patient centered communication. However, there is little research on how to effectively implement patient centered communication into pre-licensure training for physical therapists.

**Methods:**

Thirty Doctor of Physical Therapy (DPT) students took part in an educational intervention that consisted of one 4-h didactic teaching session and three 1-h experiential learning sessions. Prior to the first session, students performed an examination of a standardized patient with chronic low back pain and were assessed on psychologically informed physical therapy (PIPT) adherent behaviors via a rating scale. Students also completed the Pain Attitudes and Beliefs Scale (PABS-PT). After the last experiential session, students evaluated another standardized patient and were reassessed on PIPT adherent behaviors. Students retook the PABS-PT and qualitative data was also collected.

**Results:**

After the educational intervention, students had positive changes in their pain attitudes and belief scores indicating a stronger orientation toward a psychosocial approach to patient care (*p* < 0.05). Additionally, after the intervention, students showed improvements in their adherence to using PIPT behaviors in their simulated patient interactions (*p <* 0.05). Qualitatively, students reported a high acceptability of the educational intervention with common themes indicating improved confidence with treating and communicating with complex patients.

**Conclusion:**

Students had attitudes and beliefs shift towards a more psychosocial orientation and demonstrated improved PIPT behaviors in simulated patient interactions after a brief educational intervention. Future research should investigate best practices for implementation of psychologically informed physical therapy for licensed clinicians.

## Background

The burden of treating chronic pain conditions is felt on healthcare systems around the world [1]. In the United States, over 30% of adults experience persistent pain and more than half of adults report pain daily [2]. One way this increased burden can be met is to address psychosocial aspects of musculoskeletal pain. Psychologically informed practice can deliver psychosocially oriented care through the integration of biopsychosocial interventions into patient management. Psychologically informed interventions differ from traditional interventions by directly addressing patient pain beliefs, attitudes, emotions, and behaviors with the ultimate goal of improving patient function [3].

Physical therapy training has traditionally been focused on the biomedical model of health and illness, however, it is now widely recognized that biopsychosocial influences play a significant role in musculoskeletal prognoses [4]. Clinical practice guidelines for low back pain have emphasized the importance of incorporating psychosocial treatment approaches into routine physical therapy care, but this process has proven to be challenging with multiple studies showing that guideline-adherence is widely variable [5–8]. The incorporation of psychologically informed practice, in particular, has met multiple implementation barriers. A systematic review by Holopainen et al. investigated physical therapists’ perceptions of learning and implementing biopsychosocial interventions for musculoskeletal conditions and found that while there was a shift towards biopsychosocial care, inadequate training was a common reason for lack of confidence with implementation of psychosocial interventions [9]. Psychologically informed training for physical therapists is widely variable with trainings taking place individually or in groups, lasting from 10 to 150 h. Some trainings include single workshops while others include ongoing mentoring and learning support well past the initial didactic components [9]. This variability in training could account for some of the inconsistency with psychologically informed treatment outcomes [10].

Physical therapists’ perceptions about learning and delivering psychologically informed practice offer insight as to why its use is so variable. Nielsen et al. [11] found that a combination of ongoing learning with weekly mentor feedback was critical to successful delivery of psychologically informed treatments. However, even with ongoing interaction and mentor feedback, some physical therapists reported that they did not feel qualified to handle patients with challenging emotional needs. Physical therapists reported they did not feel confident exploring patients’ emotional distress and stated that while communication was important in understanding a patient’s perspective, they lacked any formal training in how to do this [12].

A potential solution to address these practice barriers and improve guideline adherence for low back pain care is to incorporate psychologically informed practice skills into professional training. The 2011 Institute of Medicine’s (now the National Academy of Medicine) report on pain highlighted the deficit of pain education in professional training programs and called for the inclusion of standardized pain information across health professions [13]. More recently, a 2019 National Academy of Medicine report highlighted the importance of nonpharmacological approaches to pain management and stated that clinicians still receive inadequate pre and post licensure training about non-pharmacological pain treatments, including those that are psychologically informed [14]. Specific to physical therapy training, a 2012 survey of accredited Doctor of Physical Therapy (DPT) Programs in the United States found that the average amount of time spent on pain education was 31 h (range 5–115 h) and 39% of respondents believed their students received inadequate education on pain management [15]. While this is a clear improvement over 2001 when the average amount of time spent on pain information was four hours [16], practicing physical therapists still report feeling ill-prepared to handle patients with complex pain [17]. Future directions for pain education have called for going beyond didactic content and adding more experiential training to focus on addressing the complex nuances of nonpharmacological pain treatments like building therapeutic alliance, reducing the perceived threat of pain, conceptualizing pain beliefs, and promoting self-efficacy [18].

An important component of psychologically informed practice is patient centered communication [3] however, there is little research on how to effectively implement patient centered communication into routine physical therapist’ practice. Therefore, the purpose of this study was to investigate if an educational intervention changes DPT student attitudes and beliefs about biopsychosocial approaches to patient care and improves adherence to psychologically informed communication.

## Methods

### Overview

A pre-post study design was used to evaluate an educational intervention on incorporating psychologically informed behaviors into physical therapy practice. The intervention was evaluated through use of a fidelity scale during a simulated patient case, a self-report questionnaire, and qualitative data.

### Participants

All first and second year Doctor of Physical Therapy (DPT) students (*n* = 148) were invited via email to participate in a research study to investigate the effectiveness of an educational program on students’ ability to recognize the importance of psychologically informed physical therapy (PIPT) and implement PIPT behaviors when interviewing a patient. Most Doctor of Physical Therapy curriculums in the United States are comprised of 2 years of didactic/classroom content and 1 year of off-site clinical rotations. Therefore, first and second year students were invited to participate as part of their pre-clinical training, as well as due to logistical considerations. Students were informed that they could choose to participate by returning the email to the study investigator, letting her know in person, or attending the first educational session. The educational sessions took place outside of standard curricular time and participation had no impact on student grades. There were no incentives for participation. One hundred forty-eight DPT students were invited and thirty students (20% response rate) initially chose to participate.

### Measures

Three measures were used to evaluate the program: a questionnaire on pain attitudes and beliefs to measure any changes toward a biopsychosocial approach to pain treatment, a PIPT rating scale to measure PIPT adherent behaviors, and a questionnaire to gather student reactions to the educational sessions. These measures are described in more detail below:

#### Pain attitudes and beliefs

The Pain Attitudes and Beliefs Scale for Physiotherapists [19] (PABS-PT) is a 19-item tool for the assessment of healthcare providers’ attitudes and beliefs about treatment approaches for non-specific musculoskeletal pain. Evidence suggests that providers’ attitudes and beliefs about a patient’s pain experience will influence their treatment choices [20]. The PABS-PT assesses whether a provider is more inclined to use a biomedical approach or a biopsychosocial approach. The biomedical approach suggests that all signs and symptoms related to pain are caused by physical pathology and treatment decisions will be guided by finding damaged tissues [21]. The biopsychosocial approach suggests that psychological and social factors contribute to the development and persistence of complaints [22] and treatment decisions are guided by identifying and addressing relevant psychological and social influences. The most recent version of the PABS-PT consists of two factors that distinguish between a biomedical (10 items) and a biopsychosocial treatment orientation (9 items). The older, 20-item version of the Pain Attitudes and Beliefs Scale [23] (PABS) was used for this study with 10 biomedical questions (range score 10–60) and 10 biopsychosocial questions (range score 10–60). Providers rate statements about treatment preferences on a 6-point Likert scale ranging from ‘totally disagree’ (1) to ‘totally agree’ (6). Higher scores on each subscale indicate a stronger biomedical or biopsychosocial treatment orientation. For example, a student whose behavioral score changed from a 25 to a 50 indicates a stronger biopsychosocial orientation, whereas, a student whose biomedical score changed from a 45 to a 15 a weaker biomedical treatment approach. Additionally, these scores are determined independently of each other so a student could be high on both subscales; indicating strong biopsychosocial and biomedical orientations.

#### PIPT adherent behaviors

A PIPT rating scale was developed to rate physical therapist behaviors and communication style when interacting with patients with persistent pain [24]. This scale originated in the Department of Veteran’s Affairs as a program evaluation tool and was appropriate for use for student assessment because both interventions targeted increased use of psychologically informed communication. The components of the rating scale include five domains that are based on aspects of psychologist-developed interventions such as cognitive-behavioral therapy, motivational interviewing, and Acceptance and Commitment Therapy. These psychologist-developed interventions are already standardized for large-scale implementation and feature fidelity-rating scales that include both content and style. While physical therapists demonstrate increased awareness of the need for including fidelity checks when implementing rehabilitation interventions, content is typically emphasized and the importance of communication style is overlooked. The specific domains on the PIPT rating scale are interpersonal effectiveness, autonomy, collaboration/self-efficacy, active listening, and positive expectations. The scale is designed to capture the PIPT adherent behaviors using a checklist to evaluate how often the therapist exhibits the behavior on a 0–4 scale with 0 indicating that the behavior was not observed to 4 where the behavior was extensively integrated throughout the treatment session.

#### Student reactions

A Qualtrics questionnaire with 25 questions was developed for this study to gather feedback from participants about their experience with the educational intervention. There were 14 questions developed using themes [25] related to participants’ experience with meeting learning objectives (three questions-utility judgements), assessment of the learning materials (two questions-utility judgements), application of the material into practice (one question-utility judgements), and overall engagement in the sessions (eight questions-affective reactions). The development of these 14 questions were based on learners’ perceptions of the training and fell into the “reaction criteria” level of Kirkpatrick’s model for assessment in Higher Education [25]. These were assessed on a 5-point Likert scale with higher scores indicating agreement. There were also five open-ended questions to gather qualitative data about participants’ perceived barriers and facilitators to using PIPT in their future practice and their overall impressions of the educational intervention.

### Study procedures

**Fig. 1:**
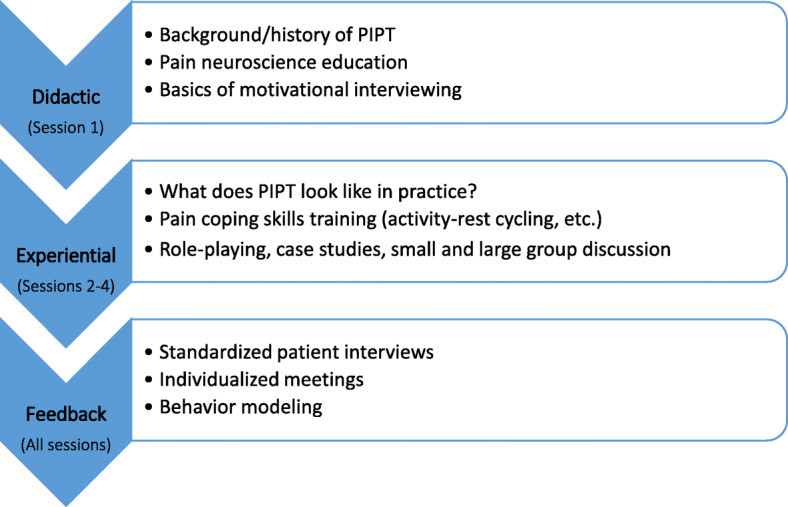
Components of PIPT Educational Intervention

The educational intervention consisted of one 4-h didactic session and three 1-h follow-up experiential learning sessions over 4 weeks. The didactic session consisted of information about the origin and definition of psychologically informed practice, how to screen for psychological risk factors [26], pain neuroscience education [27], and an introduction to PIPT adherent behaviors (interpersonal effectiveness, autonomy, collaboration/self-efficacy, active listening, and positive expectations). Figure 1 shows the flow of the training.

The three experiential learning sessions consisted of students working in groups (3–4 people) to discuss patient case studies, role-playing, and modeling of each of the PIPT adherent behaviors. After small group learning, the large group reconvened for discussion and feedback. Further details about the case studies used for role-playing are in Table 1.

**Table 1:** Specific Case Studies and Student Goals for Behaviors During Experiential Learning Sessions

Session	One		Two		Three
Behavior	Interpersonal effectiveness	Autonomy	Collaboration/Self-efficacy	Active listening	Positive expectations
Patient	35-year-old, pregnant woman who is currently smoking with low back pain.	65-year-old with 10-year history of low back pain. Exhibits catastrophizing and fear of movement.	40-year-old with 2-year history of shoulder pain after a fall at work. Exhibits fear of movement with any overhead motions.	50-year-old with multiple painful body parts and a diagnosis of fibromyalgia. Has had a bad experience with PT.	65-year-old with bilateral knee pain. Patient has been told he needs double knee replacements but cannot have surgery until he loses 20lbs.
Goal	Subjective interview ending with 2–3 value-based goals for treatment plan.	Use shared-decision making to formulate home exercise plan.	Work with patient to come to a decision about gentle movement using shared decision-making and values-based goal setting.	Interview patient and provide 2 simple reflections and 1 complex reflection.	Interview patient and provide 1–2 recommendations on where to start with exercises/weight loss plan by using tenets of positive expectations.

Prior to the first educational session, students performed an examination of a standardized patient with chronic low back pain and elevated pain catastrophizing and fear of movement. During this simulated patient session, students were assessed on their use of PIPT adherent behaviors via the PIPT rating scale. Students were not given information about their performance rating. During this session, students also completed the PABS-PT. After the last educational session, students evaluated another standardized patient with chronic low back pain and were reassessed on PIPT behaviors. They also completed the qualitative survey and retook the PABS-PT.

### Analysis

Descriptive statistics and analyses were performed with Microsoft Excel (2016). Specifically, paired-samples t tests were performed to determine if there were significant changes in the students’ orientation away from a biomedical treatment approach, (scores on biomedical subscale would decrease) and/or toward a biopsychosocial treatment approach, (scores on biopsychosocial scale would increase) and to compare the means for the PIPT adherent behaviors. This statistical approach was chosen because the pre-test scores approximated a normal distribution and paired samples t-tests are appropriate for within-subject analyses of this sample size [28, 29].

Directed content analysis [30] using NVivo 12 coding software was used for student reactions. Data (i.e. open-ended questions) was organized in response to the questions contained in the student reactions questionnaire. Two team members (LB and KB) independently examined the information for salient (i.e., recurring and/or important) emergent and a priori themes (or categories and main topics/concepts) with a focus on implementation barriers and facilitators. Disagreement with recurring and/or important themes between LB and KB were reconciled with a third team member (BE).

## Results

Twenty-two students completed the pre and post simulated patient evaluations and twenty-one students completed both the pre and post PABS-PT. Demographic information and pre to post intervention summary statistics are reported in Tables 2 and 3, respectively. The mean difference in scores was 4.48 for the biomedical scale (*p <* 0.001) with an effect size of 0.88, providing an indication that the educational intervention was associated with a decrease in the students’ orientation toward biomedical treatments. There was not a mean difference in scores for the biopsychosocial scale (*p* > 0.05). For the PIPT adherent behaviors, the mean difference in scores was − 1.125 (*p* < 0.001) with an effect size of − 2.30 providing an indication that the intervention was associated with an improvement in PIPT adherent behaviors.

**Table 2:** Descriptive Data for Sample

Variable	Total Sample (*n* = 30)
Year in school	63% 2nd year DPT students
Sex (% female)	90%
Learning objectives met (% agree)	90%
Assessment of learning material (% agree)	96%
Applicability of the material (% agree)	94%
Engagement in the sessions (% agree)	100%

**Table 3:** Pre-Intervention and Post-Intervention PABS-PT and PIPT Adherent Behaviors Mean Domain Changes

Domain	Pre-Intervention Mean (sd)	Post-Intervention Mean (sd)	t	Effect Size (Absolute Value)	p
Biomedical	33.24 (sd = 4.81)	28.76 (sd = 7.57)	4.01	0.88	< 0.001
Biopsychosocial	55.19 (sd = 4.19)	56.14 (sd = 3.14)	−1.23	0.27	0.233
PIPT behaviors	2.02 (sd = 0.54)	3.14 (sd = 0.39)	−10.80	2.30	< 0.001

### Student reactions to the educational intervention

Table 2 shows the percent agreement with each of the assessment areas from the student reaction questionnaire. Identified themes from the open-ended questions are included in Table 4 and the full list of questions from the Student Reactions Questionnaire are included in the supplementary material. For perceived benefits of the educational intervention, students reported improved confidence and communication skills. For perceived PIPT implementation challenges, comfort level and time were the two most common barriers. Themes from two of the open-ended questions (areas for improvement of educational intervention and negative aspects of educational intervention) included the desire for more opportunities to practice PIPT strategies, as well as more feedback from the instructor.

**Table 4:** Sample of Questions, Themes, and Illustrative Quotes from Student Reactions Questionnaire with the Richest Free Text Responses

	Q1: Benefits of Intervention	Q2: Perceived Implementation Challenges	Q3: Areas for Improvement of Intervention
**Themes**	*Improved confidence and communication*	*Comfort level* *and time*	*More practice and* *more feedback*
Student Quotation Supporting Theme	“I feel more equipped to help patients feel empowered with their treatment and want to view it as a PT-patient partnership rather than expert-patient relationship.” “I feel that I will now have a greater ability to communicate with all different types of patients and their emotions/coping with their injuries and pain.”	“I don’t want to force the patient to talk about something if they don’t want to, so I struggle with wanting to dig deeper but only on the patient’s terms.” “The amount of time we normally spend with patients could limit therapists’ ability to use this type of interviewing/communication strategy with patients.”	“I wish we had more sessions!...I normally dread role playing exercises, but was pleasantly surprised by how much I enjoyed them and engaged in them. I wish we had more sessions to practice.” “Possibly providing us with more structured feedback about our mock patient interview and what, if anything, we improved upon after PIPT.”
Number of responses/word count	18/676 words	18/524 words	16/473 words

## Discussion

Training physical therapists in PIPT has been a recent topic of interest in the literature [9, 31], however prior studies have focused on licensed physical therapists. Therefore, there is limited literature investigating this issue in pre-licensure physical therapists [32]. For example, this is the first study that we are aware of that used a fidelity rating scale and standardized patients to assess pre and post score changes for DPT students. The students who participated in the four-week PIPT educational intervention had favorable changes in PIPT adherent behaviors, as well as a decrease in their biomedical orientation for pain treatments. These changes were consistent with the goals of the educational intervention. However, biopsychosocial scores did not show a change possibly because students already had a strong biopsychosocial orientation before starting the intervention. Interestingly, scores were high for both beliefs subscales indicating that it is likely that DPT students have orientation towards multiple treatment approaches.

The results of this study with DPT students converge with others investigating brief interventions for improving licensed physical therapists psychologically informed treatment skills. Beneciuk and George [33] conducted a two-phase study in which one group of physical therapists received stratified care training that included 8 h of psychologically informed practice content while physical therapists in the other group received standard care training. The stratified care group had decreased biomedical scores (− 4.5 ± 2.5 points, *d* = 1.08) and increased biopsychosocial (+ 5.5 ± 2.0 points, *d* = 2.86) scores [33]. Similarly, Jacobs et al. [34] showed that a seven-hour training session had a large effect on decreasing biomedical scores (*d* = 0.93) and increasing psychosocial scores (*d* = 0.89) among outpatient physical therapists. These studies are different from the current results perhaps because licensed physical therapists had more biomedically focused training prior to taking the psychologically informed course, which may have resulted in larger changes, overall.

In this study, DPT students had high baseline biopsychosocial scores which may have resulted in a ceiling effect for changes in the PABS-PT. The high baseline scores could be due to the exposure of existing biopsychosocial treatment approaches in the DPT curriculum or previous knowledge about pain science. A course in psychology is a required pre-requisite for admission into a DPT program in the United States. Therefore, these students had previous exposure to biopsychosocial concepts during their undergraduate degrees, although that exposure would not have been specific to management of musculoskeletal pain. Another reason for higher biopsychosocial scores could be self-selection bias where students who were interested in learning more about PIPT had higher baseline knowledge. Students and licensed physical therapists have to reconcile biomedical and biopsychosocial approaches when treating patients with persistent pain. Physical therapy students are taught many special tests, mobilization techniques, and imaging protocols designed to help the clinician “find” the root cause of pain. However, recent pain research has highlighted the complex nature of pain and has questioned the reliance on special tests and imaging to provide biomedical explanations for persistent pain. This research may also help to explain why the students in this study showed high ratings on both subscales.

Other studies with licensed physical therapists have shown parallels to themes identified by DPT students with respect to barriers and facilitators to implementing PIPT. Nielsen and colleagues [11] conducted a qualitative study to identify physical therapists’ perceptions about learning psychologically informed interventions. Themes from their intervention echoed our themes with licensed physical therapists reporting improved confidence with using psychologically informed treatments with their patients with persistent pain, concerns with having enough time to deliver the PIPT intervention, as well as the desire for more mentoring and feedback [11]. This data indicates another strong convergence in results between pre and post licensure physical therapists.

These results, along with what has already been reported for licensed physical therapists, suggests that the preparation for treating patients with complex pain should start in pre-license training. The students in this study reported a high degree of acceptability of the PIPT intervention. Incorporating PIPT training with ongoing experiential learning, mentoring, and opportunities for feedback throughout the DPT curriculum will enhance the sustainability of future PIPT interventions. Other methods for increasing sustainability could be to link the learning with clinical education experiences so that students have an opportunity to practice PIPT skills with actual patients and then receive real-time feedback about their performance. This will also allow students to practice with patients of higher complexity than a simulated patient experience can provide.

While this educational intervention did show favorable changes in increasing student confidence and ability to incorporate psychologically informed interventions with patients with persistent pain, there were several limitations. A major limitation was that there was no control group. It would have been difficult to include a control educational group during an active semester when student free time is already scarce. We would have had to create a different set of comparison educational materials and then devote additional time to debrief the control students on current best practice. Other limitations include that our participants were mostly female, we had eight students drop from the study (reasons not captured), and our low response rate to participating in the educational intervention could be indicative of self-selection bias. Additionally, the PIPT rating scale was an unvalidated rating scale with no psychometric information, the use of simulated patients did not allow for direct observation of clinical skills pre and post intervention, and the Student Reactions Questionnaire was not piloted before the intervention.

**Fig. 2:**
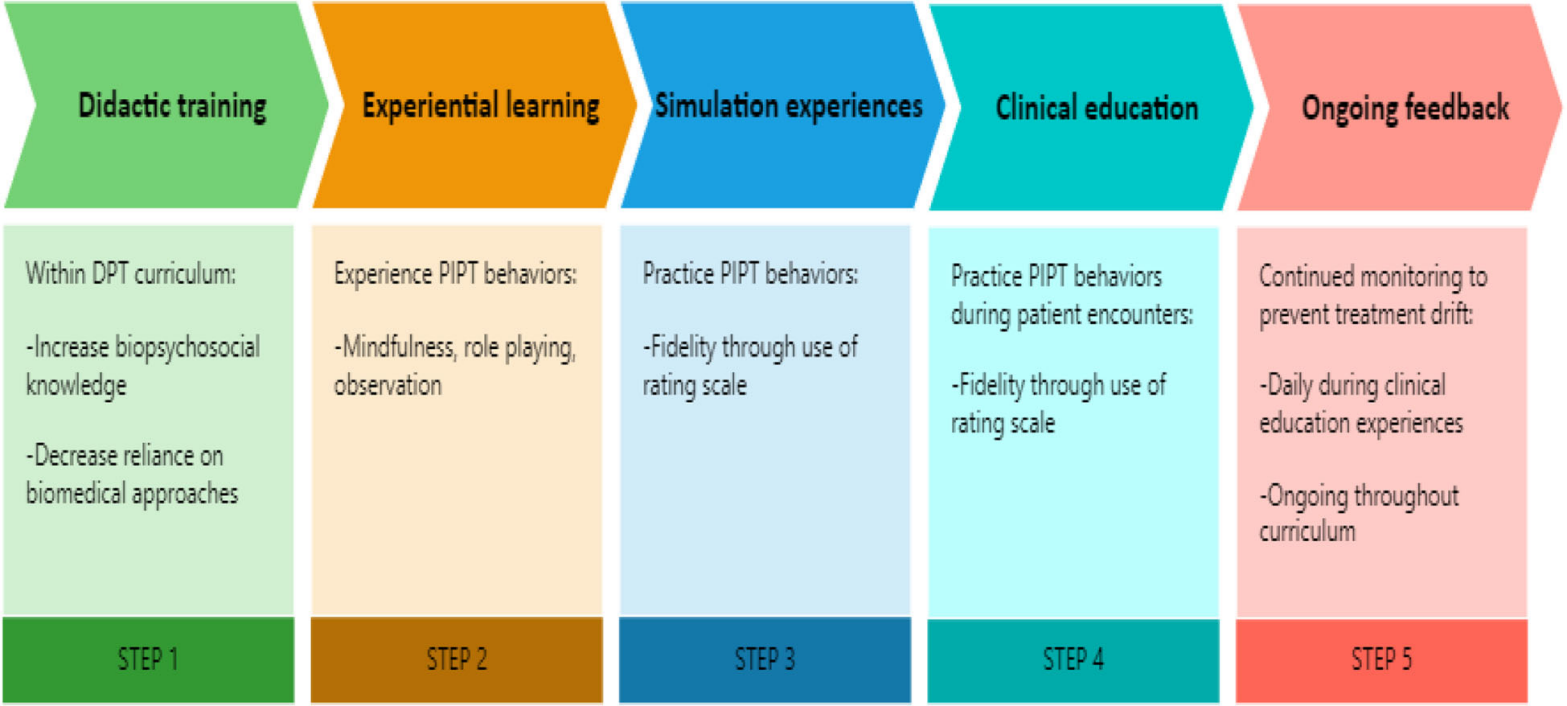
Structure for Implementation of PIPT Training into Educational Curriculum

Psychologists use fidelity-rating scales and manualized behavioral interventions as part of their daily practice (e.g. cognitive behavioral therapy, Acceptance and Commitment Therapy); however, this is not a common practice for physical therapists. Manualizing psychologically informed interventions and using a fidelity-rating tool could help to overcome some of the implementation barriers of PIPT, especially with practicing clinicians where time to learn a new skill is usually limited to a weekend course. The use of a fidelity scale could be a way to push physical therapists beyond possessing high biopsychosocial beliefs to demonstrating PIPT-adherent behaviors in practice. Use of a treatment fidelity scale may also be a way to enhance clinical education. Students could introduce the scale to their clinical instructors and both the instructor and student could enhance their learning through ongoing feedback. Figure 2 shows a possible structure for future PIPT educational implementation efforts. This is presented for pre-license training but could be adapted to train licensed physical therapists.

## Conclusion

This brief PIPT educational intervention showed favorable changes for pre-license students by decreasing biomedical treatment beliefs and increasing psychologically-informed behaviors during simulated patient interactions. The intervention was highly acceptable to students and themes that were identified converged with those from studies involving licensed physical therapists. This study showed that psychologically informed skills important for management complex pain conditions can be taught in professional training programs. This study also offers a framework for future educational implementation in other program settings.

## Supplementary information

**Additional file 1.**

## Data Availability

The datasets used and/or analyzed during the current study are available from the corresponding author on reasonable request.

## Abbreviations

DPT: Doctor of Physical Therapy
PIPT: Psychologically informed physical therapy
PABS-PT: Pain Attitudes and Beliefs Scale
