# Supplementary material for: Introduction of a psychologically informed educational intervention for pre-licensure physical therapists in a classroom setting
Source: BMC Med Educ. 2020 Oct 23;20:382. doi: 10.1186/s12909-020-02272-5 (PMC7583179; doi:10.1186/s12909-020-02272-5)
Supplement: Supplementary file 1 — Additional file 1. [file 12909_2020_2272_MOESM1_ESM.docx]

Student reaction survey to PIPT educational program

This survey asks for your opinions about the educational program you have just completed on Psychological Informed Physical Therapy practice.

Please select one response for each statement below:

Start of Block: Program objectives:

Q1 I understood the learning objectives.

- Strongly disagree (1)
- Somewhat disagree (2)
- Neither agree nor disagree (3)
- Somewhat agree (4)
- Strongly agree (5)

Q2 I was able to relate each of the learning objectives to the learning I achieved.

- Strongly disagree (1)
- Somewhat disagree (2)
- Neither agree nor disagree (3)
- Somewhat agree (4)
- Strongly agree (5)

Q3 I was appropriately challenged by the material.

- Strongly disagree (1)
- Somewhat disagree (2)
- Neither agree nor disagree (3)
- Somewhat agree (4)
- Strongly agree (5)

End of Block: Program objectives:

Start of Block: Course Materials:

Q4 I found the course materials easy to navigate.

- Strongly disagree (1)
- Somewhat disagree (2)
- Neither agree nor disagree (3)
- Somewhat agree (4)
- Strongly agree (5)

Q5 I felt that the course materials will be essential for my success.

- Strongly disagree (1)
- Somewhat disagree (2)
- Neither agree nor disagree (3)
- Somewhat agree (4)
- Strongly agree (5)

End of Block: Course Materials:

Start of Block: Content relevance:

Q6 I will be able to immediately apply what I learned.

- Strongly disagree (1)
- Somewhat disagree (2)
- Neither agree nor disagree (3)
- Somewhat agree (4)
- Strongly agree (5)

End of Block: Content relevance:

Start of Block: Facilitator knowledge:

Q7 My learning was enhanced by the knowledge of the facilitator.

- Strongly disagree (1)
- Somewhat disagree (2)
- Neither agree nor disagree (3)
- Somewhat agree (4)
- Strongly agree (5)

Q8 My learning was enhanced by the experiences shared by the facilitator.

- Strongly disagree (1)
- Somewhat disagree (2)
- Neither agree nor disagree (3)
- Somewhat agree (4)
- Strongly agree (5)

End of Block: Facilitator knowledge:

Start of Block: Facilitator delivery:

Q9 I was engaged during the session.

- Strongly disagree (1)
- Somewhat disagree (2)
- Neither agree nor disagree (3)
- Somewhat agree (4)
- Strongly agree (5)

Q10 It was easy for me to get actively involved in the session.

- Strongly disagree (1)
- Somewhat disagree (2)
- Neither agree nor disagree (3)
- Somewhat agree (4)
- Strongly agree (5)

Q11 I was comfortable with the pace of the session.

- Strongly disagree (1)
- Somewhat disagree (2)
- Neither agree nor disagree (3)
- Somewhat agree (4)
- Strongly agree (5)

Q12 I was comfortable with the duration of the session.

- Strongly disagree (1)
- Somewhat disagree (2)
- Neither agree nor disagree (3)
- Somewhat agree (4)
- Strongly agree (5)

Q13 I was given ample opportunity to have my questions answered.

- Strongly disagree (1)
- Somewhat disagree (2)
- Neither agree nor disagree (3)
- Somewhat agree (4)
- Strongly agree (5)

Q14 I was given ample opportunity to practice the skills I learned in this session.

- Strongly disagree (1)
- Somewhat disagree (2)
- Neither agree nor disagree (3)
- Somewhat agree (4)
- Strongly agree (5)

End of Block: Facilitator delivery:

Start of Block: Benefits and challenges

Please answer the following questions in the space provided.

What do you see as the biggest challenges to using the principles of Psychologically Informed Physical Therapy in clinical practice? Please describe.

____________________________________________________________________________

____________________________________________________________________________

____________________________________________________________________________

____________________________________________________________________________

Do you feel you have benefitted from participating in this learning program?

- Yes (1)
- No (2)

If yes, please list the benefits you believe you have gained by participating in this program?

____________________________________________________________________________

____________________________________________________________________________

____________________________________________________________________________

____________________________________________________________________________

Were there any negative aspects for you related to participating in this learning program?

- Yes (1)
- No (2)

If yes, please list the negative aspects of the program below.

____________________________________________________________________________

____________________________________________________________________________

____________________________________________________________________________

____________________________________________________________________________

End of Block: Benefits and challenges:

Start of Block: General:

How could we improve this educational program for future students?

____________________________________________________________________________

____________________________________________________________________________

____________________________________________________________________________

____________________________________________________________________________

Do you have any other comments about the learning program?

____________________________________________________________________________

____________________________________________________________________________

____________________________________________________________________________

____________________________________________________________________________

End of Block: General:

Start of Block: Completion page

***Thank you for providing your feedback on the PIPT learning experience.***
